# Prenatal Exposure to Phthalates and Childhood Body Size in an Urban Cohort

**DOI:** 10.1289/ehp.1408750

**Published:** 2015-06-12

**Authors:** Michelle M. Maresca, Lori A. Hoepner, Abeer Hassoun, Sharon E. Oberfield, Stephen J. Mooney, Antonia M. Calafat, Judyth Ramirez, Greg Freyer, Frederica P. Perera, Robin M. Whyatt, Andrew G. Rundle

**Affiliations:** 1 Division of Pediatric Endocrinology, Diabetes and Metabolism, Department of Pediatrics, New York-Presbyterian Hospital, Columbia University, New York, New York, USA; 2 Columbia Center for Children’s Environmental Health, Department of Environmental Health Sciences, and; 3 Department of Epidemiology, Mailman School of Public Health, Columbia University, New York, New York, USA; 4 Division of Laboratory Sciences, National Center for Environmental Health, Centers for Disease Control and Prevention, Atlanta, Georgia, USA

## Abstract

**Background:**

Phthalate exposures are hypothesized to increase obesity; however, prior research has been largely cross-sectional.

**Objective:**

We evaluated associations between prenatal phthalate exposures and body mass index (BMI) at child ages 5 and 7 years.

**Methods:**

Nine metabolites of six phthalates—di(2-ethylhexyl) phthalate (DEHP), di-n-octyl-, di-iso-butyl-, di-n-butyl-, butylbenzyl-, and diethyl phthalates—were measured in spot urine samples collected from pregnant African-American and Dominican women during their third trimester, and from their children at ages 3 and 5 years. To reduce multiple comparison issues, we initially used principal component analysis (PCA) to identify major patterns of natural log (ln)-transformed metabolite concentrations. Height and weight were assessed at ages 5 and 7 years, and fat mass and waist circumference at age 7. Linearized generalized estimating equation analyses related maternal component scores to child anthropometric outcomes at ages 5 (n = 326) and 7 (n = 330) years.

**Results:**

PCA identified a DEHP component and a non-DEHP component. In boys, higher maternal non-DEHP, but not DEHP, component scores were associated with lower BMI z-score (β = –0.30; 95% CI: –0.50, –0.10, n = 156), lower fat percentage (β = –1.62; 95% CI: –2.91, –0.34, n = 142), and smaller waist circumference (β = –2.02; 95% CI: –3.71, –0.32, n = 124). No significant associations with anthropometric outcomes were seen in girls (for BMI z-score, β = 0.07; 95% CI: –0.18, 0.31, n = 181). Interactions between sex and non-DEHP component association with outcomes were statistically significant (p < 0.01).

**Conclusions:**

Contrary to hypotheses, prenatal non-DEHP phthalate exposures were associated with lower BMI z-score, waist circumference, and fat mass in boys during early childhood.

**Citation:**

Maresca MM, Hoepner LA, Hassoun A, Oberfield SE, Mooney SJ, Calafat AM, Ramirez J, Freyer G, Perera FP, Whyatt RM, Rundle AG. 2016. Prenatal exposure to phthalates and childhood body size in an urban cohort. Environ Health Perspect 124:514–520; http://dx.doi.org/10.1289/ehp.1408750

## Introduction

Certain endocrine-disrupting chemicals (EDCs), including phthalates, are hypothesized to be obesogens and contribute to the obesity epidemic (Grün and Blumberg 2009; Heindel 2003; Newbold et al. 2008). Phthalates are a class of EDCs with ubiquitous human exposure, commonly found in plastics, fragrances, cosmetics, and building materials (Sathyanarayana 2008; Silva et al. 2004). Phthalates have anti-androgenic and weakly estrogenic properties, and have been found to increase adipogenesis *in vitro* and in some animal studies (Biemann et al. 2012; Hao et al. 2012; Harris et al. 1997; Jobling et al. 1995; Lovekamp and Davis 2001; Rengarajan et al. 2007; Takeuchi et al. 2005). However, other animal studies have shown either a decrease or no significant change in adipose weight after prenatal phthalate exposure (Boberg et al. 2008; Casals-Casas et al. 2008; Casals-Casas and Desvergne 2011). Cross-sectional studies using National Health and Nutrition Examination Survey (NHANES) data found that urinary concentrations of five phthalate metabolites [monobenzyl phthalate (MBzP), mono(2-ethyl-5-oxohexyl) phthalate (MEOHP), mono(2-ethyl-5-hydroxyhexyl) phthalate (MEHHP), monoethyl phthalate (MEP), mono-*n*-butyl phthalate (MBP)] correlated positively with body mass index (BMI) in adult males (Hatch et al. 2008; Stahlhut et al. 2007), and similarly MEP correlated positively with BMI in adolescent females (Hatch et al. 2008). However, an inverse correlation was observed between mono(2-ethylhexyl) phthalate (MEHP) and BMI in adolescent and adult females (Hatch et al. 2008). In cross-sectional analyses among children, low-molecular-weight phthalates were positively associated with BMI and odds of overweight and obesity, but only among non-Hispanic blacks (Trasande et al. 2013a). A study by Teitelbaum et al. (2012) found that higher urinary concentrations of MEP and molar sum of MEP, MBP, and mono-isobutyl phthalate (MiBP) measured at age 6–8 years were associated with higher BMI a year later among overweight children. However, previous studies have been limited by cross-sectional designs or short follow-up periods, and have not evaluated potential effects of exposures during critical developmental periods of fetal and early newborn life.

Using the Columbia Center for Children’s Environmental Health (CCCEH) longitudinal birth cohort, we conducted prospective analyses of maternal urinary phthalate metabolite concentrations and child body size at ages 5 and 7 years, with further assessment of associations between body size and childhood phthalate exposures at ages 3 and 5 years. We hypothesized that prenatal phthalate exposures would be associated with higher BMI *z*-scores (age- and sex-adjusted BMI), greater fat mass, and larger waist circumferences and that associations would be stronger among boys due to phthalates’ possible anti-androgenic effect.

## Methods

*Participant selection and data collection.* The children in this study are enrolled in the CCCEH longitudinal birth cohort study’s Obesity Project, which has been described extensively elsewhere (Perera et al. 2003; Rundle et al. 2012). Recruitment took place between 1998 and 2006 through prenatal clinics in northern Manhattan, at New York-Presbyterian Hospital and Harlem Hospital Center. Inclusion criteria were pregnant women between 18 and 35 years of age, who self-identified as either African American or Dominican, and resided in Northern Manhattan or the South Bronx in New York City for at least 1 year before pregnancy. Exclusion criteria were current smoking at time of recruitment, illicit drug use, or having a diagnosis of HIV, hypertension, or diabetes. During the third trimester of pregnancy, a trained bilingual interviewer collected sociodemographic information from participants. Infants’ sex and birth weight were obtained from medical records following delivery. As described previously, at ages 5 and 7 years the children’s heights were measured on a SECA wall-mounted stadiometer to the nearest 0.1 cm (SECA), age 5 weight was measured to the nearest 0.1 kg using a Detecto Cardinal 750 digital scale (Cardinal Scale Manufacturing Company), and at age 7 years total weight and fat mass were measured using a Tanita scale (Model BC-418; Tanita Corporation of America). Measurements were taken while wearing light clothes and no shoes (Rundle et al. 2012). BMI *z*-scores and percentiles were calculated using the Centers for Disease Control and Prevention (CDC) SAS macro; obesity was defined as BMI for age and sex > 95th percentile (CDC 2004). Waist circumference was measured midway between the lower rib margin and iliac crest using a nonstretching tape measure (Rundle et al. 2012).

The institutional review boards of Columbia University Medical Center and the CDC approved this study. All mothers gave informed consent at enrollment, and all children at age 7 years assented to participate.

*Measurement of urinary phthalate metabolites.* Spot urine samples were collected from the mothers during the third trimester of pregnancy, and from the children at ages 3 and 5 years using a urine cup supplied by the CDC. The specific gravity of the urine samples was quantified at room temperature at Columbia University with a handheld refractometer (PAL 10-S; Atago). Samples were frozen at –80°C immediately after collection and shipped frozen to the CDC for analysis. Using solid phase extraction–high performance liquid chromatography/isotope dilution tandem mass spectrometry (Silva et al. 2007), urine samples were analyzed for metabolites of di(2-ethylhexyl) phthalate (DEHP), which are MEHP, MEHHP, MEOHP, and mono(2-ethyl-5-carboxypentyl) phthalate (MECPP); and for metabolites of di-isobutyl phthalate (DiBP), di-*n*-butyl phthalate (DnBP), butylbenzyl phthalate (BBzP), diethyl phthalate (DEP), and di-*n*-octyl phthalate (DOP), which are, respectively, MiBP, MBP, MBzP, and MEP, and mono(3-carboxypropyl) phthalate (MCPP), a nonspecific metabolite of several phthalates.

*Data analysis.* All analyses were performed using PASW® Statistics 21 (SPSS®) and SAS version 9.3 (SAS institute Inc.). The value of the limit of detection (LOD)/2 was assigned to phthalate metabolite concentrations below the LOD [MEHP: 54 samples (LOD, 0.5–1.2 ng/dL), MCPP: 18 samples (LOD, 0.16–1 ng/dL), MiBP (LOD, 0.2–1.038 ng/dL), and MBzP (LOD, 0.11–1 ng/dL); 1 sample each]. As recommended by the CDC, reported concentrations of MEP and MBzP were multiplied by 0.66 and 0.72, respectively, to correct for impurity in the analytic standards which led to overestimation of the concentrations of these metabolites (CDC 2014). Urinary phthalate metabolite concentrations were natural log (ln)–transformed for analyses.

Correlations between ln-transformed concentrations of urinary metabolites were assessed using Pearson product moment correlation analyses (see Supplemental Material, Table S1). Because of medium to strong correlations between urinary phthalate metabolite concentrations (0.34–0.99, *p* < 0.001 for all correlations), and to reduce multiple comparisons, the initial statistical analytical plan used principal component analysis (PCA) to generate summary measures of phthalate exposure patterns from the urinary metabolite data (Hastie et al. 2009; Kleinbaum et al. 1988). PCA with varimax rotation was applied to ln-transformed maternal and child (at ages 3 and 5 years) urinary metabolite concentrations. PCA is a data reduction tool that describes variability among multiple correlated, observed variables in terms of a fewer number of unobserved variables, referred to as components, and has been used to identify patterns of exposure from complex chemical mixtures (Arif and Shah 2007; Bengraïne and Marhaba 2003; Bergh et al. 2011; Billionnet et al. 2012; Burstyn 2004; Dominici et al. 2010; Guo et al. 2004; Lampa et al. 2012; Zitko 1994). Component scores were calculated for the mothers and the children at ages 3 and 5 years, and represent how closely the metabolites in a subject’s urine sample conform to identified metabolite patterns. Because PCA results depend on the correlation structure within a particular data set, the correlation and PCA analyses were also applied to 2003–2004 NHANES phthalate urinary metabolite data to ascertain whether the components identified in this study data were consistent with nationally representative data (CDC 2014). Phthalate urinary metabolite concentration data for MEHP, MEHHP, MECPP, MEOHP, MCPP, MiBP, MBP, MBzP, and MEP from women ages 15–45 years who took part in the 2003–2004 NHANES were downloaded (*n* = 595). This time period best overlaps with enrollment and urine collection in the CCCEH. The metabolite data were ln-transformed and analyzed using PCA with varimax rotation. Analyses incorporated the NHANES complex survey weights.

The molar sum of DEHP metabolite (MEHP, MEHHP, MEOHP, and MECPP) concentrations in maternal urine was also calculated.

We used a generalized estimating equation (GEE) model with a linear link function and unstructured correlation structure (Hubbard et al. 2010) to assess whether maternal urinary phthalate concentration component scores were associated with child BMI *z*-scores at ages 5 and 7 years. We used linear regression analyses to test whether maternal urinary phthalate concentration component scores were associated with child age 7 years percent body fat, fat mass index (FMI; fat mass in kilograms/height in meters squared) and waist circumference (centimeters). All models were adjusted for maternal race/ethnicity (self-reported as African American or Dominican), receipt of public assistance during pregnancy, prepregnancy obesity status (based on mother’s self-reported height and prepregnancy weight), child birth weight (continuous variable), child age in months at the time of follow-up at ages 5 and 7 years, and maternal urine specific gravity *z*-score to adjust for urine concentration because it is associated with component scores (though less strongly associated with raw metabolite concentrations). Analyses were conducted separately in boys and girls, and a model incorporating sex by component score interaction terms was fit to formally test whether associations varied by sex. Secondary analyses were undertaken when significant associations (*p* < 0.05) were identified in stratified analyses, assessing relationships between the anthropometric outcome and individual phthalate metabolites loading heavily for the component.

A series of sensitivity analyses were conducted to assess potential confounding effects by phthalate exposures at child age 3 and 5 years, stability of the results to different model specifications, and possible bias from loss to follow-up. To assess possible confounding between maternal and child phthalate exposures, we assessed correlations between the mothers’ component scores and the children’s ages 3 and 5 years component scores, and the children’s component scores were added to the GEE model described above. Possible interactions between maternal urinary phthalate component scores and age 5 versus age 7 follow-up wave were assessed by including a follow-up wave by component scores interaction term in the model. To assess possible bias due to incomplete follow-up at ages 5 and 7 years, inverse probability weights for successful assessment at each of these two waves were calculated as described previously (Curtis et al. 2007; Hernán et al. 2004; Rundle et al. 2012), and these weights were incorporated into the GEE models.

## Results

Prenatal urinary phthalate metabolite concentrations were available from 424 mothers. Geometric mean concentrations of phthalate metabolites in maternal urine samples and intercorrelations between concentrations are reported in Supplemental Material, Table S1.The correlation matrix observed for the mothers’ metabolite concentrations was very similar to that observed in NHANES (see Supplemental Material, Table S2). Cohort follow-up and urinary metabolite data availability are documented in Supplemental Material, Figure S1. From the 424 mothers with urinary phthalate data, 326 children had anthropometric outcome data at age 5 years (310 with complete covariate data), 330 had anthropometric outcome data at age 7 years (314 with complete covariate data), 303 had outcome data at both age 5 and 7 years, and 353 had outcome data at age 5 or 7 years. Among the 326 children followed to age 5, urinary metabolite data were available from 234 children at age 3 years. Among the 330 children followed to age 7, urinary metabolite data were available from 241 at age 3 and 302 at age 5 years.

Table 1 provides descriptive data for the cohort. Nineteen percent of the children were obese at age 5 and 25% were obese at age 7 years. PCA identified two components in the maternal urinary metabolite data with eigenvalues > 1 (see Supplemental Material, Figure S2), one component in the child age 3 data, and two components in the child age 5 data, representing major patterns of phthalate metabolite concentrations among participants (Table 2). The first maternal component, labeled the “DEHP” component, accounted for 61% of the variation in the data; it loaded highly for DEHP metabolites and was highly correlated with the molar sum of DEHP metabolites (*r* = 0.95). The second component, the “non-DEHP” component, accounted for an additional 16% of the variation in the data and loaded highly for non-DEHP metabolites. PCA analysis of child age 5 phthalate metabolite concentrations also identified a DEHP and non-DEHP component, but analysis of child age 3 urine revealed only a single component. PCA of NHANES phthalate metabolite data identified two components with very similar loadings to the components observed in the CCCEH mothers’ data (see Supplemental Material, Table S3).

**Table 1:** Characteristics of mothers and children with urinary phthalate measurements and childhood anthropometric outcomes [*n* (%) or mean ± SD unless otherwise specified].

Characteristic	Total enrolled cohort (*n* = 424)	With age 5 anthropometric data (*n* = 326)	With age 7 anthropometric data (*n* = 330)
Sex of child
Female	219 (52)	178 (55)	173 (52)
Male	205 (48)	148 (45)	157 (48)
Ethnicity
African American	139 (33)	119 (36.5)	119 (36)
Dominican	285 (67)	207 (63.5)	211 (64)
Maternal prepregnancy obesity (BMI > 30)
Yes	85 (20)	70 (22)	71 (22)
No	328 (77)	248 (76)	251 (76)
Missing	11 (3)	8 (2.5)	8 (2)
Maternal receipt of public assistance
Yes	182 (56)	141 (43)	140 (42)
No	238 (43)	183 (56)	188 (57)
Missing	4 (1)	2 (1)	2 (1)
Child obesity^*a*^
Yes	NA	63 (19)	82 (25)
No	NA	263 (79)	248 (75)
Mean birth weight (g)	3,375 (± 472)	3,372 (± 481)	3,383 (± 489)
Mean BMI *z*-score	NA	0.58 (± 1.43)	0.83 (± 1.16)
Median BMI percentile (interquartile range)	NA	72.00 (35.44, 92.83)	81.76 (54.09, 95.13)
Mean percent body fat (*n* = 322)	NA	NA	24.38 (± 6.34)
Mean fat mass index (*n* = 322)	NA	NA	4.62 (± 2.33)
Waist circumference (cm) (*n *= 318)	NA	NA	59.91 (± 8.66)
DEHP component score	0.00 (± 1.00)	0.03 (± 0.99)	0.00 (± 0.99)
Non-DEHP component score	0.00 (± 1.00)	0.04 (± 0.98)	0.01 (± 1.00)
Abbreviations: BMI, body mass index; DEHP, di(2-ethylhexyl) phthalate; NA, not applicable. ^***a***^Child obesity defined as BMI ≥ 95th percentile for age and sex according to the CDC growth charts.			

**Table 2:** Rotated component-loading weights for phthalate urinary metabolite concentrations in CCCEH mothers and children.

Phthalate metabolite	Maternal		Child age 3	Child age 5	
	DEHP component^*a*^	Non-DEHP component^*a*^	Phthalate component^*b*^	DEHP component^*c*^	Non-DEHP component^*c*^
MEHP	0.88	0.23	0.81	0.90	0.15
MEHHP	0.94	0.31	0.95	0.92	0.34
MECPP	0.92	0.29	0.93	0.90	0.31
MEOHP	0.92	0.35	0.95	0.91	0.38
MCPP	0.39	0.68	0.87	0.49	0.60
MiBP	0.26	0.79	0.83	0.34	0.79
MBP	0.19	0.89	0.90	0.41	0.83
MBzP	0.21	0.77	0.78	0.25	0.76
MEP	0.23	0.60	0.63	0.07	0.66
Abbreviations: DEHP, di(2-ethylhexyl) phthalate; MBP, mono-*n*-butyl phthalate; MBzP, monobenzyl phthalate; MCPP, mono(3-carboxypropyl) phthalate; MECPP, mono(2-ethyl-5-carboxypentyl) phthalate; MEHHP, mono(2-ethyl-5-hydroxyhexyl) phthalate; MEHP, mono(2-ethylhexyl) phthalate; MEOHP, mono(2-ethyl-5-oxohexyl) phthalate; MEP, monoethyl phthalate; MiBP, mono-isobutyl phthalate. ^***a***^The DEHP component explains 61% and the non-DEHP component explains 16% of the total variance in the metabolite data. ^***b***^The phthalate component explains 73% of the total variance in the metabolite data. ^***c***^The DEHP component explains 63% and the non-DEHP component explains 14% of the total variance in the metabolite data.					

Table 3 displays associations between maternal phthalate metabolite component scores and child BMI *z*-scores at ages 5 and 7 years. Prenatal DEHP component scores were not significantly associated with BMI *z*-scores at ages 5 and 7. In all three models, prenatal non-DEHP component score was significantly associated with lower BMI *z*-scores in boys only. Associations of child BMI *z*-scores with prenatal DEHP and non-DEHP component scores were similar after adjustment for component scores from the children’s urine samples collected at age 3 (model 2) or at age 5 years (model 3). In model 1 (which did not include the children’s urine metabolite concentrations), the interaction between sex and non-DEHP component score was statistically significant [β = –0.30; 95% confidence interval (CI): –0.50, –0.10 in boys compared with β = 0.07; 95% CI: –0.18, 0.31 in girls; *p* for interaction = 0.003]. Similarly, higher maternal non-DEHP component scores were significantly associated with lower percent body fat, lower FMI, and smaller waist circumference at age 7 years in boys but not girls (Table 4). For example, for fat percentage, β = –1.62; 95% CI: –2.91, –0.34 for boys and β = 0.62; 95% CI: –0.64, 1.88 for girls; *p* for interaction = 0.002. There were no associations with DEHP component score and percent body fat, FMI, or waist circumference in boys or girls. Secondary analyses examined associations between BMI *z*-score, fat percentage, FMI, and waist circumference, and individual phthalates that loaded highly for the non-DEHP component. Generally, the associations between the individual metabolites and anthropometric outcomes did not reach statistical significance, but revealed similar patterns of inverse associations in boys (Table 5). The molar sum of the DEHP metabolites was also analyzed and, as with the DEHP component score, had no associations with BMI *z*-score, fat percentage, FMI, or waist circumference.

**Table 3:** Associations between maternal prenatal urinary phthalate metabolite component scores and child BMI *z*-scores at ages 5 and 7 years.

Phthalate component score	Model 1^*a*^			Model 2^*b*^			Model 3^*c*^		
	Girls (*n* = 181) β (95% CI) *p*-value	Boys (*n *= 156) β (95% CI) *p*-value	*p*-int^*d*^	Girls (*n* = 127) β (95% CI) *p*-value	Boys (*n* = 113) β (95% CI) *p*-value	*p*-int^*d*^	Girls (*n* = 154) β (95% CI) *p*-value	Boys (*n* = 124) β (95% CI) *p*-value	*p*-int^*d*^
Prenatal DEHP component score	–0.10 (–0.29, 0.09) 0.30	–0.08 (–0.30, 0.13) 0.45	ND^*e*^	–0.16 (–0.42, 0.09) 0.21	–0.06 (–0.34, 0.22) 0.66	ND^*e*^	–0.12 (–0.31, 0.08) 0.25	0.00 (–0.24, 0.24) 0.99	ND^*e*^
Prenatal non-DEHP component score	0.07 (–0.18, 0.31) 0.58	–0.30 (–0.50, –0.10) 0.003	0.003	0.16 (–0.17, 0.48) 0.35	–0.31 (–0.56, –0.07) 0.01	0.003	0.13 (–0.14, 0.41) 0.34	–0.30 (–0.54, –0.06) 0.01	0.001
Age 3 phthalate component score	NA	NA	NA	0.02 (–0.34, 0.37) 0.92	0.00 (–0.29, 0.29) 0.99	ND^*e*^	NA	NA	NA
Age 5 DEHP component score	NA	NA	NA	NA	NA	NA	–0.01 (–0.20, 0.23) 0.91	–0.13 (–0.38, 0.13) 0.33	ND^*e*^
Age 5 non-DEHP component score	NA	NA	NA	NA	NA	NA	–0.12 (–0.37, 0.14) 0.36	–0.34 (–0.58, –0.10) 0.01	ND^*e*^
Abbreviations: DEHP, di-(2-ethylhexyl) phthalate; NA, not applicable; ND, not done. ^***a***^GEE-based analyses were used with BMI *z*-score data from ages 5 and 7 years combined; the β coefficients reflect the average difference in BMI *z*-score per unit difference in component score. Model 1 adjusts for age (in months) at time of measurement, maternal prepregnancy obesity, birth weight, maternal race/ethnicity, maternal receipt of public assistance during pregnancy, and urinary specific gravity. The DEHP and non-DEHP component score variables are entered into the same regression model and thus the results are mutually adjusted. ^***b***^Model 2 additionally adjusts for child age 3 urinary metabolite concentration component score. ^***c***^Model 3 additionally adjusts for child age 5 urinary metabolite concentration DEHP and non-DEHP component scores. ^***d***^*p* for interaction with sex. ^***e***^Interaction test not performed because stratified analyses did not suggest heterogeneity of effect sizes by child sex.									

**Table 4:** Associations*^a^* between maternal prenatal urinary phthalate component scores and child body composition and waist circumference outcomes at age 7 years.

Phthalate component	Percent body fat			Fat mass index			Waist circumference		
	Girls (*n* = 164) β (95% CI) *p*-value	Boys (*n* = 142) β (95% CI) *p*-value	*p*-int^*b*^	Girls (*n* = 164) β (95% CI) *p*-value	Boys (*n* = 142) β (95% CI) *p*-value	*p*-int^*b*^	Girls (*n* = 154) β (95% CI) *p*-value	Boys (*n* = 124) β (95% CI) *p*-value	*p*-int^*b*^
Prenatal DEHP component score	–0.14 (–1.19, 0.92) 0.80	–0.31 (–1.56, 0.94) 0.63	ND^*c*^	–0.01 (–0.42, 0.39) 0.96	–0.11 (–0.56, 0.34) 0.63	ND^*c*^	–0.11 (–1.46, 1.25) 0.88	–0.65 (–2.26, 0.97) 0.43	ND^*c*^
Prenatal non-DEHP component score	0.62 (–0.64, 1.88) 0.33	–1.62 (–2.91, –0.34) 0.01	0.002	0.34 (–0.15, 0.82) 0.17	–0.50 (–0.96, –0.04) 0.03	0.003	1.20 (–0.43, 2.84) 0.15	–2.02 (–3.71, –0.32) 0.02	0.0002
Abbreviations: DEHP, di(2-ethylhexyl) phthalate; ND, not done. ^***a***^All analyses adjust for age (in months) at time of measurement, maternal prepregnancy obesity, birth weight, maternal race/ethnicity, receipt of public assistance during pregnancy, and urinary specific gravity. The DEHP and non-DEHP component score variables are entered into the same regression model and thus the results are mutually adjusted. The β coefficient reflects the average difference in the outcome variable per unit difference in component score. ^***b***^*p* for interaction with sex. ^***c***^Interaction test not performed because stratified analyses did not suggest heterogeneity of effect sizes by child sex.									

**Table 5:** Associations*^a^* between maternal urinary phthalate metabolites and child anthropometric outcomes.

Maternal phthalate urinary metabolite	BMI *z*-score at ages 5 and 7^*b*^		Percent body fat at age 7		FMI at age 7		Waist circumference at age 7	
	Girls (*n* = 181) β (95% CI) *p*-value	Boys (*n* = 156) β (95% CI) *p*-value	Girls (*n* = 164) β (95% CI) *p*-value	Boys (*n* = 142) β (95% CI) *p*-value	Girls (*n* = 164) β (95% CI) *p*-value	Boys (*n* = 142) β (95% CI) *p*-value	Girls (*n* = 154) β (95% CI) *p*-value	Boys (*n* = 124) β (95% CI) *p-*value
Molar sum of DEHP metabolites	–0.08 (–0.25, 0.09) 0.37	–0.09 (–0.28, 0.11) 0.39	–0.13 (–1.09, 0.84) 0.80	–0.39 (–1.57, 0.79) 0.51	–0.03 (–0.40, 0.34) 0.88	–0.13 (–0.55, 0.29) 0.54	–0.13 (–1.37, 1.12) 0.84	–0.65 (–2.16, 0.87) 0.40
MCPP	0.10 (–0.17, 0.37) 0.46	–0.24 (–0.47, –0.01) 0.04	0.08 (–1.07, 1.23) 0.89	–0.63 (–1.86, 0.60) 0.32	0.14 (–0.30, 0.58) 0.52	–0.19 (–0.63, 0.25) 0.40	0.70 (–0.76, 2.16) 0.34	–0.88 (–2.51, 0.76) 0.29
MiBP	0.02 (–0.18, 0.22) 0.86	–0.17 (–0.36, 0.01) 0.06	0.78 (–0.33, 1.88) 0.17	–0.97 (–2.10, 0.17) 0.09	0.35 (–0.08, 0.77) 0.11	–0.29 (–0.70, 0.11) 0.16	1.12 (–0.34, 2.58) 0.13	–1.18 (–2.66, 0.31) 0.12
MBP	0.11 (–0.12, 0.34) 0.33	–0.22 (–0.44, 0.00) 0.05	–0.52 (–0.72, 1.76) 0.41	–1.05 (–2.26, 0.15) 0.09	0.32 (–0.15, 0.80) 0.18	–0.34 (–0.78, 0.09) 0.12	0.85 (–0.76, 2.47) 0.30	–1.34 (–2.91, 0.23) 0.10
MBzP	–0.04 (–0.18, 0.10) 0.56	–0.16 (–0.31, –0.01) 0.04	0.03 (–0.81, 0.87) 0.95	–0.75 (–1.64, 0.15) 0.10	0.03 (–0.30, 0.35) 0.87	–0.18 (–0.50, 0.14) 0.26	0.10 (–1.03, 1.19) 0.86	–0.70 (–1.87, 0.48) 0.24
MEP	0.02 (–0.15, 0.19) 0.83	–0.04 (–0.19, 0.10) 0.56	0.33 (–0.61, 1.27) 0.49	–0.86 (–1.78, 0.07) 0.07	0.12 (–0.24, 0.49) 0.50	–0.32 (–0.65, 0.01) 0.06	0.63 (–0.56, 1.83) 0.30	–1.27 (–2.46, –0.08) 0.04
Abbreviations: BMI, body mass index; FMI, fat mass index; MBP, mono-*n*-butyl phthalate; MBzP, monobenzyl phthalate; MCPP, mono(3-carboxypropyl) phthalate; MEP, monoethyl phthalate; MiBP, mono-isobutyl phthalate. ^***a***^All analyses adjust for age (in months) at time of measurement, maternal prepregnancy obesity, birth weight, maternal race/ethnicity, receipt of public assistance during pregnancy, and urinary specific gravity. Metabolite concentrations were ln-transformed for analyses. The β coefficient reflects the average difference to in the outcome variable per unit difference in the ln-transformed metabolite concentration. ^***b***^GEE-based analyses were used with BMI *z*-score data from ages 5 and 7 years combined.								

Maternal phthalate component scores were not associated with child phthalate component scores (data not shown), and adjustment for child age 3 or age 5 component scores did not alter the associations (Table 3). However, among boys, the child age 5 non-DEHP component score was inversely associated with BMI *z*-score at ages 5 and 7 years on GEE analysis. Tests of interaction between follow-up wave and maternal urinary metabolite component scores were not statistically significant, and there were no significant differences between the component scores of children who were lost to follow-up and children with follow-up (data not shown).

## Discussion

Contrary to our hypothesis that phthalates would be positively associated with body size, our results suggest that prenatal exposures to non-DEHP phthalates, as reflected by higher non-DEHP component scores in maternal urine, are associated with lower BMI *z*-score, fat mass, and waist circumference in boys at ages 5 and 7 years in our study population. Prenatal exposure to DEHP, as measured by the DEHP component score and molar sum of DEHP metabolites, was not associated with anthropometric outcomes in children through age 7 years. Relationships between maternal urinary phthalate metabolite concentrations and child BMI *z*-score did not appear to be confounded by the child’s urinary phthalate component scores at age 3 years or the DEHP and non-DEHP component scores at age 5 years.

The inverse association between non-DEHP component scores and anthropometric outcomes among boys was unexpected; given experimental evidence that phthalates have endocrine-disrupting anti-androgenic or estrogenic effects, we expected a positive, not inverse, association with increased fat mass in boys. Nonetheless, the results do support the hypothesis that phthalates can interfere with the processes of adipogenesis and fat metabolism. We caution against interpreting that phthalates are beneficial in reducing obesity risk, however. Continued ongoing cohort follow-up will reveal whether this inverse association persists, and further analyses will help determine the clinical significance of the association.

The longitudinal cohort design of this study is a major strength because prior cross-sectional designs may be biased by diet. Higher food intake leads to both increased body size as well as higher exposures to phthalate contaminants in food, making it difficult to infer causality in cross-sectional analyses (Boas et al. 2010; Colacino et al. 2010; Hatch et al. 2008; Serrano et al. 2014; Stahlhut et al. 2007; Trasande et al. 2013b). Teitelbaum et al. (2012) conducted the only other published prospective study, with urinary phthalate metabolites measured at mean age 7.3 years and BMI data collected on average 1 year later. Among overweight children, higher MEP and molar sum of MEP, MBP, and MiBP were associated with higher BMI; however, no significant associations were observed overall (Teitelbaum et al. 2012). In contrast, we did not observe any statistically significant positive associations between BMI *z*-score and maternal or child urine phthalate metabolite concentrations.

PCA has been used to assess patterns of exposure from complex chemical mixtures, and may further be particularly advantageous for studying urinary metabolite concentrations (Billionnet et al. 2012; Burstyn 2004; Dominici et al. 2010; Pan et al. 2007). Correlations between metabolite concentrations are expected to reflect exposures to a single parent compound, exposures to several parent compounds with similar exposure routes, and/or the result of parent compound metabolism by the same or similarly induced metabolic pathways; these scenarios are aligned with PCA approaches to construct latent variables reflecting patterns of metabolite concentrations. This was clearly represented by identification of a primary component loading highly for DEHP metabolites, almost certainly reflecting DEHP exposure. Additionally, PCA was useful for identifying a second component that loaded heavily for the non-DEHP metabolites and predicted anthropometric outcomes, whereas analyses of each of the non-DEHP metabolites separately produced results that were generally of borderline statistical significance. A limitation of PCA is that characterization of the components is dependent on the specific study sample data, and observed component scores may not be widely generalizable. However, we found that when applied to NHANES data, PCA found very similar components and component loadings as observed in CCCEH mothers (see Supplemental Material, Tables S1–S3).

Some limitations of this study should be considered. To our knowledge, this is the first longitudinal birth cohort study examining associations between prenatal phthalate exposure and subsequent childhood anthropometric outcomes, and these results must be confirmed in other populations and cohorts. Due to the exploratory nature of this initial work, *p*-values were not corrected for multiple comparisons, and the significance of the findings may be overestimated. Another limitation was availability of only one spot urine sample per mother, and due to intraindividual variation in urinary metabolite concentrations, exposure was likely categorized with some error (Braun et al. 2012; Frederiksen et al. 2013; Preau et al. 2010; Teitelbaum et al. 2008). Additionally, this cohort consists exclusively of urban African-American and Dominican families, almost all from low-income backgrounds, which limits the generalizability of the work. Further, data on physical activity, diet, and other potential confounders were not available for these subjects; thus bias from residual confounding may exist. However, although diet and physical activity most certainly affect anthropometric outcomes, it is difficult to accurately measure these factors in young children (Bell et al. 2013; Burrows et al. 2010; Chinapaw et al. 2010; de Lauzon-Guillain et al. 2012). Last, the sample size available for sex stratified analyses was limited.

## Conclusions

Although cross-sectional studies suggest that phthalate exposures may increase obesity predisposition, our longitudinal birth cohort study did not find any statistically significant positive association between child anthropometric outcomes and maternal prenatal urine phthalate metabolite concentrations. Conversely, there were inverse associations between maternal non-DEHP component score and BMI *z*-score, body fat percentage, FMI, and waist circumference in boys only. However, phthalate exposures have been linked to several poor health outcomes in children (Sathyanarayana 2008; Whyatt et al. 2014), and we caution against interpreting these results to suggest that phthalates are beneficial in reducing obesity risk. Further cohort follow-up will delineate whether negative associations between prenatal exposures and body size in boys persist into adolescence, or if new associations emerge.

## Supplemental Material

(435 KB) PDF
