# Supplementary material for: Prenatal Exposure to Phthalates and Childhood Body Size in an Urban Cohort
Source: Environ Health Perspect. 2015 Jun 12;124(4):514–20. doi: 10.1289/ehp.1408750 (PMC4829975; doi:10.1289/ehp.1408750)

Environ Health Perspect
DOI: 10.1289/ehp.1408750

Note to Readers: *EHP* strives to ensure that all journal content is accessible to all readers. However, some figures and Supplemental Material published in *EHP* articles may not conform to 508 standards due to the complexity of the information being presented. If you need assistance accessing journal content, please contact ehp508@niehs.nih.gov. Our staff will work with you to assess and meet your accessibility needs within 3 working days.

Supplemental Material

Prenatal Exposure to Phthalates and Childhood Body Size in an Urban Cohort

Michelle M. Maresca, Lori A. Hoepner, Abeer Hassoun, Sharon E. Oberfield, Stephen J. Mooney, Antonia M. Calafat, Judyth Ramirez, Greg Freyer, Frederica P. Perera, Robin M. Whyatt, and Andrew G. Rundle

Table of Contents

Table S1. Descriptive statistics and correlations[a] between maternal urinary phthalate metabolite concentrations.

Table S2. Correlations between phthalate urinary metabolite concentrations for women ages 15-45 in NHANES 2003-2004.

Table S3. Rotated component loading weights for phthalate urinary metabolite concentrations for women ages 15 to 45 in NHANES 2003-2004.

Figure S1. Cohort follow-up. Figure S1 depicts follow-up of the birth cohort to ages 3, 5 and 7. Sample sizes in boxes attached to arrows reflect the number of children followed from each follow-up point to a subsequent follow-up point. Sample sizes in circles reflect the number of children lost to follow-up after a specific follow-up point.

Figure S2. Component plot for principal component analysis of CCCEH maternal urinary phthalate metabolites concentrations.

Table S1. Descriptive statistics and correlations[a] between maternal urinary phthalate metabolite concentrations.

	Geometric Mean (SD)[b]	MEHP	MEHHP	MECPP	MEOHP	MCPP	MIBP	MBP	MBZP	MEP	Molar Sum of DEHP metabolites	DEHP Component
**MEHP**	4.91 (4.21)											
**MEHHP**	22.03 (3.56)	0.86										
**MECPP**	39.04 (3.08)	0.81	0.94									
**MEOHP**	18.30 (3.48)	0.85	0.99	0.95								
**MCPP**	1.98 (2.82)	0.44	0.56	0.55	0.59							
**MIBP**	8.81 (2.87)	0.44	0.48	0.46	0.52	0.52						
**MBP**	37.58 (2.83)	0.40	0.46	0.44	0.49	0.60	0.73					
**MBZP**	13.40 (3.74)	0.38	0.44	0.41	0.47	0.53	0.56	0.65				
**MEP**	164.14 (3.53)	0.34	0.39	0.39	0.40	0.45	0.40	0.50	0.34			
**Molar Sum of DEHP metabolites**	292.89 (3.24)	0.87	0.97	0.98	0.99	0.57	0.49	0.46	0.44	0.40		
**DEHP Component**	0.00 (1.00)	0.88	0.94	0.92	0.92	0.39	0.26	0.19	0.21	0.23	0.95	
**Non-DEHP Component**	0.00 (1.00)	0.23	0.31	0.29	0.35	0.68	0.79	0.89	0.77	0.60	0.31	0.00

[a]Pearson product moment correlations for natural log transformed urinary metabolite concentrations, all p-values <0.001.

[b]Values reported are geometric means and standard deviations except for the DEHP and non-DEHP components which are reported as means and standard deviations. Individual phthalate measures are in ng/ml of urine, the sum of DEHP metabolites is measured in nmol/L, and the component scores are unit less.

Table S2. Correlations[a] between phthalate urinary metabolite concentrations for women ages 15-45 in NHANES 2003-2004.

	MEHP	MEHHP	MECPP	MEOHP	MCPP	MIBP	MBP	MBZP
**MEHHP**	0.81							
**MECPP**	0.78	0.96						
**MEOHP**	0.80	0.98	0.96					
**MCPP**	0.45	0.57	0.57	0.58				
**MIBP**	0.43	0.51	0.48	0.51	0.64			
**MBP**	0.43	0.56	0.52	0.57	0.75	0.75		
**MBZP**	0.35	0.50	0.46	0.50	0.69	0.63	0.70	
**MEP**	0.20	0.23	0.23	0.24	0.33	0.40	0.42	0.33

[a]Pearson product moment correlations for natural log transformed urinary metabolite concentrations.

Table S3. Rotated component loading weights for phthalate urinary metabolite concentrations for women ages 15 to 45 in NHANES 2003-2004.

	**DEHP Component[a]**	**Non-DEHP Component[a]**
**MEHP**	0.87	0.18
**MEHHP**	0.94	0.29
**MECPP**	0.93	0.27
**MEOHP**	0.93	0.31
**MCPP**	0.40	0.75
**MIBP**	0.31	0.80
**MBP**	0.33	0.84
**MBZP**	0.28	0.79
**MEP**	0.01	0.63

[a]The DEHP Component explains 62% and the non-DEHP component explains 16% of the total variance in the metabolite data.

Figure S1. Cohort follow-up. Figure S1 depicts follow-up of the birth cohort to ages 3, 5 and 7. Sample sizes in boxes attached to arrows reflect the number of children followed from each follow-up point to a subsequent follow-up point. Sample sizes in circles reflect the number of children lost to follow-up after a specific follow-up point.

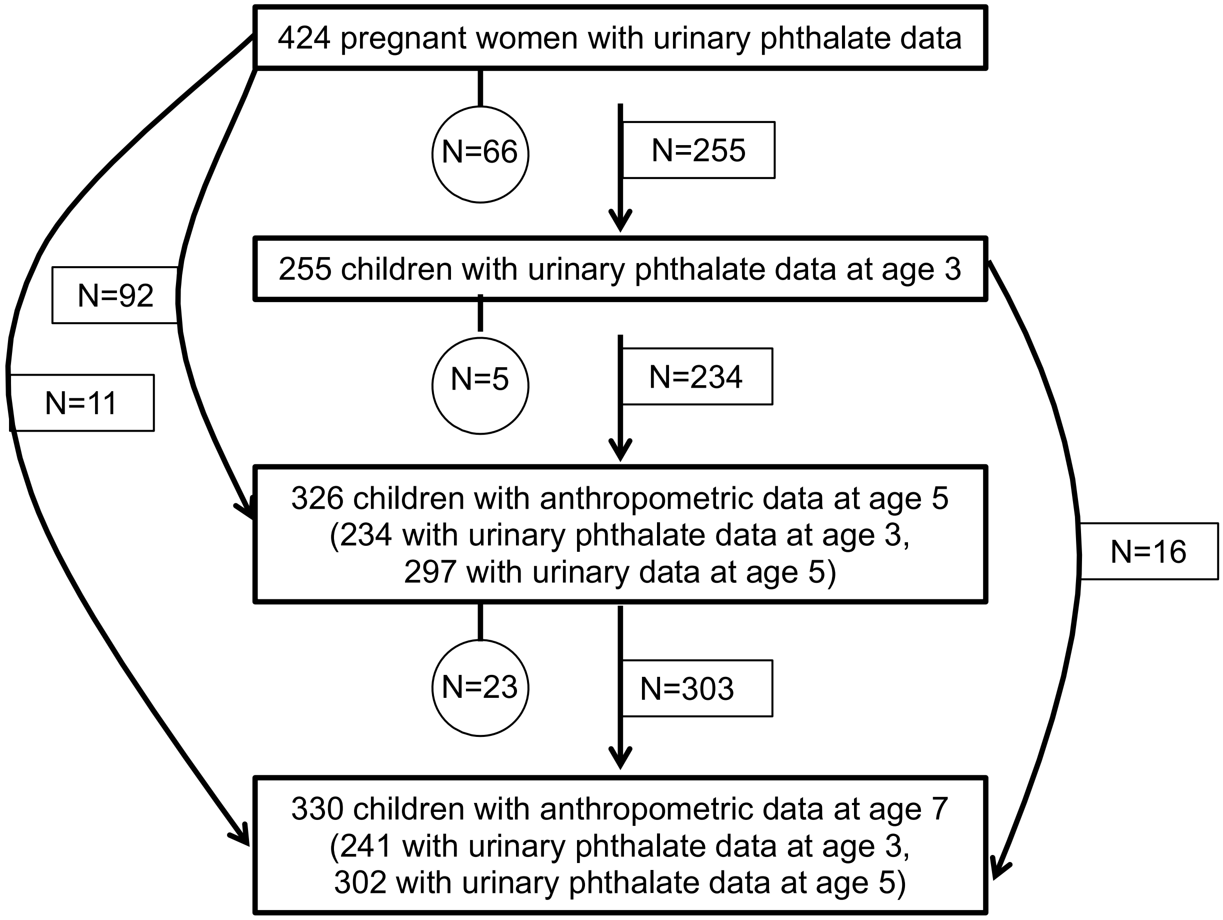

Figure S2. Component plot for principal component analysis of CCCEH maternal urinary phthalate metabolites concentrations.

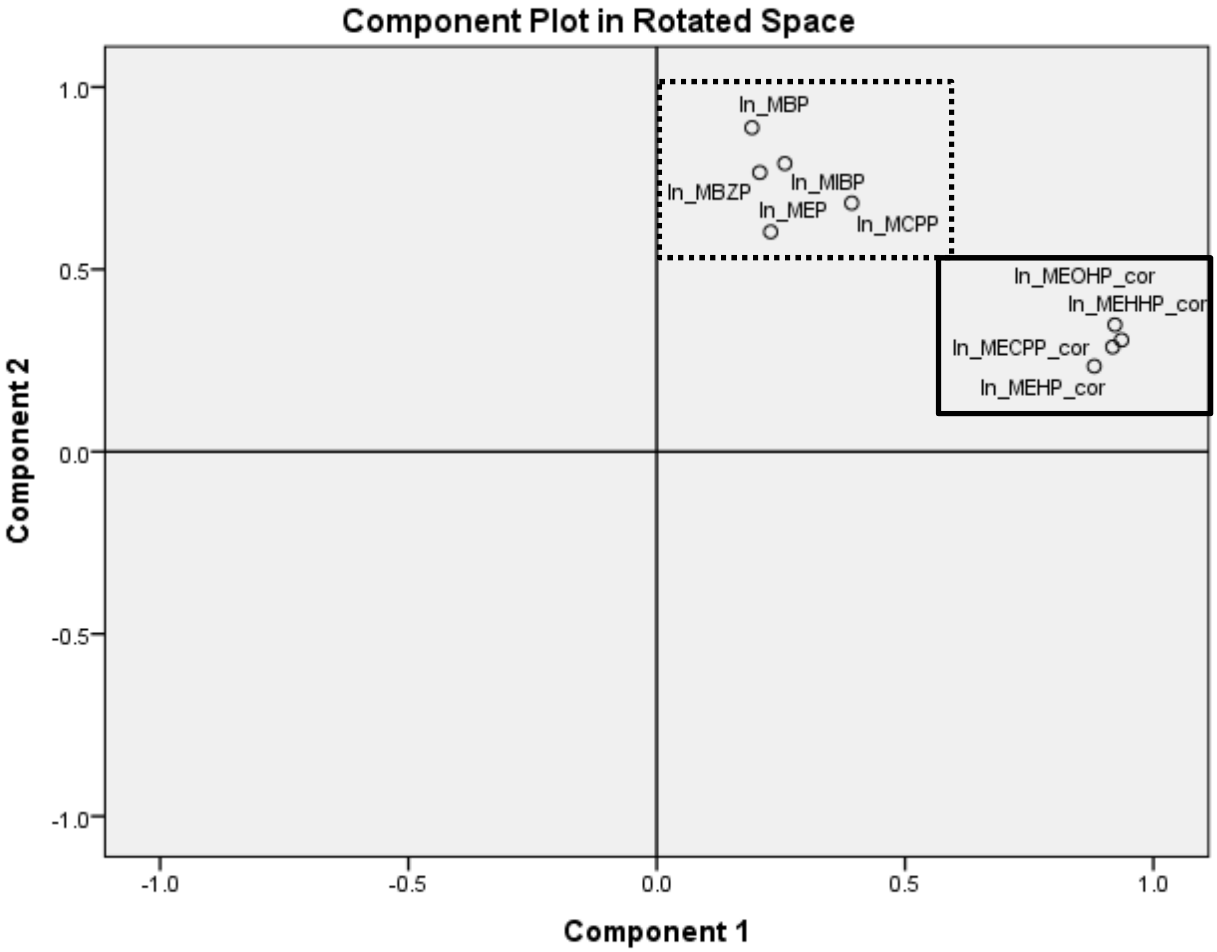

Legend:

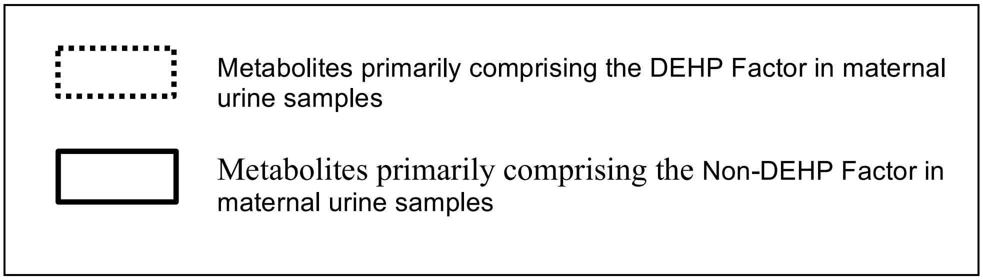

Supplement: (435 KB) PDF [file ehp.1408750.s001.acco.pdf]
